# Probing the Honey Bee Diet-Microbiota-Host Axis Using Pollen Restriction and Organic Acid Feeding

**DOI:** 10.3390/insects11050291

**Published:** 2020-05-09

**Authors:** Vincent A. Ricigliano, Kirk E. Anderson

**Affiliations:** 1 USDA-ARS, Honey Bee Breeding, Genetics, and Physiology Laboratory, Baton Rouge, LA 70820, USA; 2 USDA-ARS, Carl Hayden Bee Research Center, Tucson, AZ 85719, USA

**Keywords:** *Apis mellifera*, microbiota, microbiome, host-microbe interactions, organic acids, nutrition, rectal pads, pollen

## Abstract

Microbial metabolites are considered important drivers of diet-based microbiota influence on the host, however, mechanistic models are confounded by interactions between diet, microbiota function, and host physiology. The honey bee harbors a simple microbiota that produces organic acids as fermentation products of dietary nectar and pollen, making it a model for gut microbiota research. Herein, we demonstrate that bacterial abundance in the honey bee gut is partially associated with the anterior rectum epithelium. We used dietary pollen restriction and organic acid feeding treatments to obtain information about the role of undigested pollen as a microbiota growth substrate and the impact of bacterial fermentation products on honey bee enteroendocrine signaling. Pollen restriction markedly reduced total and specific bacterial 16S rRNA abundance in the anterior rectum but not in the ileum. Anterior rectum expression levels of bacterial fermentative enzyme gene transcripts (acetate kinase, lactate dehydrogenase, and hydroxybutyryl-CoA dehydrogenase) were reduced in association with diet-induced microbiota shifts. To evaluate the effects of fermentative metabolites on host enteroendocrine function, pollen-restricted bees were fed an equimolar mixture of organic acid sodium salts (acetate, lactate, butyrate, formate, and succinate). Organic acid feeding significantly impacted hindgut enteroendocrine signaling gene expression, rescuing some effects of pollen restriction. This was specifically manifested by tissue-dependent expression patterns of neuropeptide F and allatostatin pathways, which are implicated in energy metabolism and feeding behaviors. Our findings provide new insights into the diet-microbiota-host axis in honey bees and may inform future efforts to improve bee health through diet-based microbiota manipulations.

## 1. Introduction

Animals form a wide variety of associations with environmental and symbiotic microbes and the effects of these associations on animal physiology are a focus of considerable research interest. Insects (the largest and most diverse group of animals) are promising models to study host-microbe interactions since a range of cellular and metabolic functions are conserved throughout the animal kingdom [1]. Thus, understanding cross talk between insects and microbes could have broader applicability to other systems, including humans. Insects, and honey bees, in particular, provide unique opportunities to study complex host-microbe interactions because they tend to associate with fewer and less diverse microbes [2,3]. Insect gastrointestinal tracts harbor not only the highest density of microbes, but also the most metabolically active microbial community, or microbiota [4]. The primary metabolic functions carried out by insect microbiota are: (i) fermentation of indigestible dietary components and metabolite production that influences host energy metabolism and physiology, (ii) production of essential nutrients that are absent from the host’s food such as amino acids, (iii) salvaging of host metabolic waste products [5,6].

The honey bee (*Apis mellifera*) gut is populated with a simple but specific microbiota that influences host nutrient utilization, immunity, development, hormone signaling, and behavior [7,8,9]. While physiochemical conditions of the bee crop and midgut create a less suitable microbial environment, the hindgut harbors a large community that expresses approximately 10 ^9^ bacterial 16S ribosomal rRNA copies [10,11]. The hindgut is divided into the ileum and rectum, each with a characteristic community phenotype [10,12]. The ileum is a narrow tube connecting the midgut to the rectum and is chiefly colonized by a biofilm of two-gram-negative species: *Snodgrassella alvi* (Betaproteobacteria) and *Gilliamella apicola* (Gammaproteobacteria) [13]. The rectum accounts for most of the hindgut bacteria and is dominated by gram-positive species, including two *Lactobacillus* taxa (Firm 5 and Firm 4) and *Bifidobacterium asteroides* [12,14]. Recent studies collectively suggest that the honey bee microbiota ferments undigested pollen as a growth substrate, producing a variety of alcohols, short-chain fatty acids, and other organic acids that influence host physiology and behavior [9,15,16,17].

Due to their interface with the gut epithelium, the microbiota are poised to interact with epithelial cells and in this way convey information from the microbiota to the host [1]. Within vertebrate and invertebrate gut epithelia, endocrine (enteroendocrine) cells act as nutrient sensors, responding to environmental stimuli and secreting enteroendocrine peptides that act on the endocrine, nervous, and immune systems to mediate adaptive physiological responses [18,19,20,21,22]. Understanding differs widely with respect to different classes of these peptides (also referred to as neuropeptides) and their functional roles within a given insect species. However, neuropeptide hormones have been implicated in processes such as development, reproduction and behavior [20,23,24]. A recent survey of enteroendocrine peptide diversity in the honey bee gut identified precursors for 15 different peptide families including neuropeptide F [25], which is presently only characterized in the bee brain [26]. Accumulating evidence suggests that insect neuropeptide F (NPF), a homolog of the well-characterized mammalian neuropeptide Y, regulates several physiological processes and food-related behaviors [27]. In *Drosophila*, NPF signaling pathways are expressed in the brain and gut: upregulation of brain NPF signaling mimics starvation-induced changes in foraging behaviors [28], whereas gut NPF signaling controls germline stem cell proliferation in a mating-dependent manner [29]. Analysis of honey bee gut enteroendocrine peptide diversity also identified allatostatins [25], which were previously characterized in the bee brain to modulate learning [30]. In other insects, these neuropeptides inhibit juvenile hormone signaling [31], reduce food intake [32], and influence foraging behaviors [33]. However, the roles of NPF and allatostatin signaling in the honey bee gut are currently unknown.

Given that neuropeptides and gut hormones target the same cell membrane receptors (G protein-coupled receptors, GPCRs), the two messenger roles typically converge on the same functions due to shared cellular transduction systems [19,24]. Enteroendocrine cells respond to nutrients and digestive metabolites via GPCRs, leading to the release of neuropeptides [34]. Enteroendocrine cells are also stimulated by microbially-produced organic acids, which can interact directly with GPCRs [35]. Organic acids (notably the short-chain fatty acids acetate, propionate, and butyrate) are a major class of microbiota metabolites derived from the fermentation of dietary fibers [36]. These metabolites have profound and conserved effects on host physiology [37,38]. Some effects of the honey bee microbiota are suggested to be mediated by organic acids [9,17], although there is limited evidence as to how. Microbiota metabolism reflects environmental conditions such as substrate presence, co-bionts, and the physiochemical environment of distinct gut compartments [39]. Therefore, mechanistic understandings of microbiota effects require consideration of community abundance, metabolic traits, host physiology, and diet context. In this study, we tested the hypothesis that the majority of the fermentative bacterial community in the bee hindgut is localized to the anterior rectum, a putative host-microbe interface. Dietary pollen restriction was used to quantify the *in vivo* role of undigested pollen as a bacterial growth and fermentation substrate. Finally, we tested the hypothesis that bacterial fermentative metabolites can modulate honey bee enteroendocrine signaling by feeding pollen-restricted bees an equimolar cocktail of organic acid sodium salts (acetate, lactate, butyrate, formate, and succinate).

## 2. Materials and Methods

### 2.1. Determination of Bacterial Abundance in the Honey Bee Anterior Rectum

**Figure 1:**
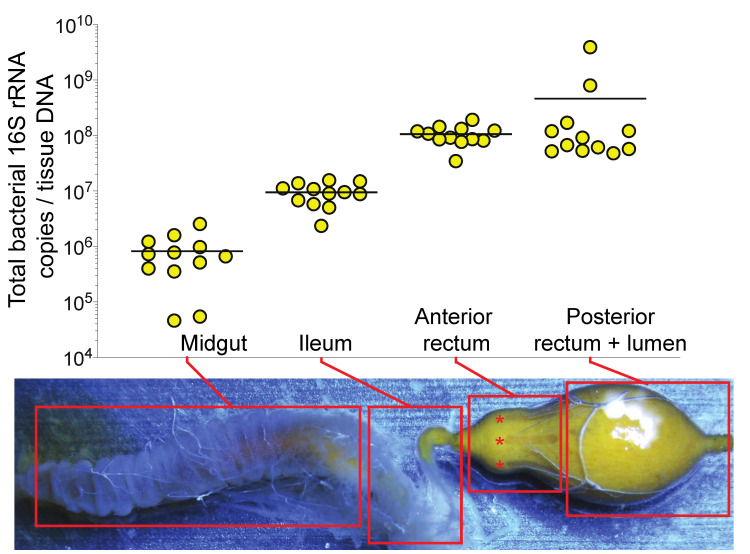
Quantification of bacterial 16S abundance in honey bee gut tissue total DNA extracts. The rectum was subsectioned into the rectal-pad containing the anterior portion and the posterior portion plus lumen contents. The anterior rectum was washed to remove the bacterial signal from the lumen. Each point represents the average 16S copies from a pool of 12 nurse-aged bees from a distinct colony (*n* = 12 colonies). Horizontal black lines represent the mean. Rectal pads are marked with asterisks *.

All experiments were conducted in July 2018 at the USDA-ARS Carl Hayden Honey Bee Research Center Tucson, Arizona, USA. Nurse-aged bees were obtained from the center of the brood nest from 12 healthy colonies. Bees were held by the thorax with sterile forceps in one hand, and an abdominal sternite was grasped with a second pair of forceps to facilitate removal of the entire digestive tract from the abdomen. From each of 12 colonies, 12 pollen-containing “nurse” guts per colony were sectioned according to the scheme outlined in Figure 1 and pooled into the following tissue types: midgut, ileum, anterior rectum, posterior rectum + lumen contents. For sectioning of the rectum, the anterior portion of the rectum (including host rectal pads, marked with an asterisk in Figure 1) was held with forceps in one hand, and the rectum was held over a sample tube and cut with microscissors to catch the posterior portion plus lumen contents (see Figure 1), then the anterior portion was added to a separate collection tube. Samples, therefore, consisted of 12 colony replicates of 12 pooled guts for each of 4 tissue types. Anterior rectum tissues were washed with 500 μl of sterile dH_2_O in a microcentrifuge tube by vortexing to remove lumen contamination. The samples were pelleted by centrifugation then the supernatant was carefully removed and discarded, leaving behind the washed anterior rectum tissues. This was repeated three times to facilitate the removal of lumen bacterial signal. Samples were transferred to 2-ml bead-beating tubes containing 0.2 g of 0.1-mm silica beads and DNA was extracted using an AllPrep PowerViral DNA/RNA Kit (Qiagen, Hilden, Germany) according to the manufacturer’s instructions. Total bacterial abundance was estimated by qPCR using universal bacterial 16S rRNA gene copies and a standard curve generated from a 10-fold serial dilution series of a plasmid DNA containing a full-length *Escheria coli* 16S rRNA gene. The qPCR results were expressed as the total number of 16S rRNA gene copies per tissue sample by multiplying by the total DNA concentration in each sample [40].

### 2.2. Experimental Setup for Pollen and Organic Acid Feeding

Newly emerged bees were sourced from brood frames of 12 healthy colonies containing late-stage pupae, from which adults emerged naturally at 35 °C and 50% relative humidity. Newly emerged bees no older than 16 h post-emergence were collected into a single large container, mixed thoroughly, and 65 individuals were placed into Plexiglass cages. Cages were subjected to one of three diet treatments: a) polyfloral bee-collected pollen plus 50% sucrose solution (w/v); b) 50% sucrose solution (w/v) only; or c) 50% sucrose solution (w/v) formulated with 10 mM each of organic acid sodium salts (acetate, lactate, butyrate, formate, and succinate; Sigma). Cage was the unit of replication for this experiment. Therefore, 16–19 cages per treatment group (pollen fed, pollen restricted, pollen restricted + organic acids) were set up and bees were sampled after 9 days onto dry ice and stored at −80 °C.

### 2.3. RNA Extraction and qPCR

Bees from the above experiment were dissected on ice and hindgut tissues were subsectioned into the ileum and anterior rectum for RNA extraction. From each cage, 12 bees were dissected into two pools containing 12 ilea or 12 anterior rectums which were collected into cold 2-ml bead-beating tubes, immediately frozen on dry ice, and stored at −80 °C for RNA extraction. RNA extractions were carried out using an AllPrep PowerViral DNA/RNA Kit (Qiagen) according to the manufactures instructions. cDNA was synthesized from 1 μg of DNaseI treated total RNA using a RevertAid First Strand cDNA Synthesis Kit (Thermo Fisher Scientific, Waltham, MA, USA). qPCR using cDNA template was carried out in triplicate using iTaq™ Universal SYBR^®^ Green Supermix (Biorad, Hercules, CA, USA) on a CFX96™ Real-Time PCR Detection System (Biorad). Cycling conditions were as follows: initial denaturation at 95 °C for 5 mins; 40 cycles with denaturation at 95 °C for 15s; and a primer-pair-specific annealing and extension temperature (Table S1) for 30 seconds. To confirm the absence of contaminating genomic DNA and primer dimers, we monitored amplification and melting curves in negative controls consisting of DNase-treated total RNA without reverse transcriptase. Relative gene expression across treatments was determined based on standardized Ct values (Δ Ct) [41] using honey bee actin as a reference gene [42].

### 2.4. Comparison of Bacterial Abundance and Fermentative Enzyme Gene Expression

Bacterial abundance was determined by qPCR and using previously published universal bacteria and species-specific 16S rRNA primers (Table S1), on cDNA synthesized hindgut tissue total RNAs. Each ileum and anterior rectum sample was screened with primers targeting the honey bee actin gene, the universal 16S rRNA, and species-specific 16S rRNAs of five bacterial groups considered core to the gut microbiota (*Lactobacillus* Firm 5, *Lactobacillus* Firm 4, *Bifidobacterium*, *Gilliamella*, and *Snodgrassella*). Normalization to host actin facilitated comparison of bacterial abundance and gene expression across treatments.

For bacterial metabolic genes, previously published primer pairs targeting enzyme transcripts belonging to specific gut taxa (*Lactobacillus Firm 5*, *Bifidobacterium*, and *Gilliamella* were used to amplify acetate kinase (*ackA*) and lactate dehydrogenase (*ldh*) [43] (Table S1). We selected two candidates bacterial hydroxybutyryl-CoA dehydrogenase (*hbd*) gene candidates based on published genome data [44] to measure expression by *Lactobacillus* Firm 4 and *Bifidobacterium* (Table S1). Relative bacterial abundance and metabolic gene expression were determined based on standardized Ct values (Δ Ct) [41] using honey bee actin as a reference gene as in [42].

### 2.5. Comparison of Honey Bee Enteroendocrine Signaling Gene Expression

Honey bee enteroendocrine signaling gene expression in the ileum and anterior rectum was measured by qPCR using the cDNA template. The profiled gene transcripts related to neuropeptide F signaling were neuropeptide F (*npf*) and neuropeptide F receptor (*snpfr*) [26] (Table S1). The profiled gene transcripts related to allatostatin signaling were allatostatin A (*Apime-ASTA*), allatostatin A receptor (*Apime-ASTA-R*) allatostatin C (*Apime-ASTC*), allatostatin CC (*Apime-ASTCC*), *Apime-ASTC/CC* receptor (*Apime-ASTC-R*) (Table S1). Relative gene expression was determined based on standardized Ct values (Δ Ct) [41] using honey bee actin as a reference gene [42].

### 2.6. Statistical Analyses

Cage (*n* = 16–19) was the experimental unit of replication for this study. The effects of diet treatments on bacterial 16S rRNA abundance, bacterial metabolic gene expression, and honey bee enteroendocrine signaling were evaluated using one-way ANOVA. Variables with deviations from normality were re-evaluated after log transformation. The Tukey HSD post hoc test was used to compare different treatment groups. Analyses were conducted in JMP v11.

## 3. Results

### 3.1. Bacterial Abundance in the Honey Bee Gut is Partially Associated with the Anterior Rectum Epithelium

To obtain an increased resolution of honey bee gut microbiota localization and abundance, nurse bee guts were sectioned into the midgut, ileum, anterior rectum, and posterior rectum plus lumen contents. Consistent with previous reports [10], the midgut and ileum featured ~10^6^ and ~10^7^ bacterial 16S rRNA DNA copies per total DNA sample, respectively (Figure 1). Subsectioning the rectum revealed that ~10^8^ 16S DNA copies per total DNA sample are detectable in the washed anterior portion and ~10^8^ 16S DNA copies in the posterior portion plus lumen contents (Figure 1). Due to this apparent localization of bacterial signal, proximity to the biofilm-containing ileum, and presence of host rectal pads (marked with asterisks in Figure 1), we chose to further examine the anterior rectum in subsequent diet manipulation experiments. Due to its proximity to the anterior rectum, the ileum was also chosen as a target tissue for further examination of bacterial abundance and gene expression.

### 3.2. Dietary Pollen Restriction Markedly Reduces Total and Specific Bacterial Abundance in the Anterior Rectum

**Figure 2:**
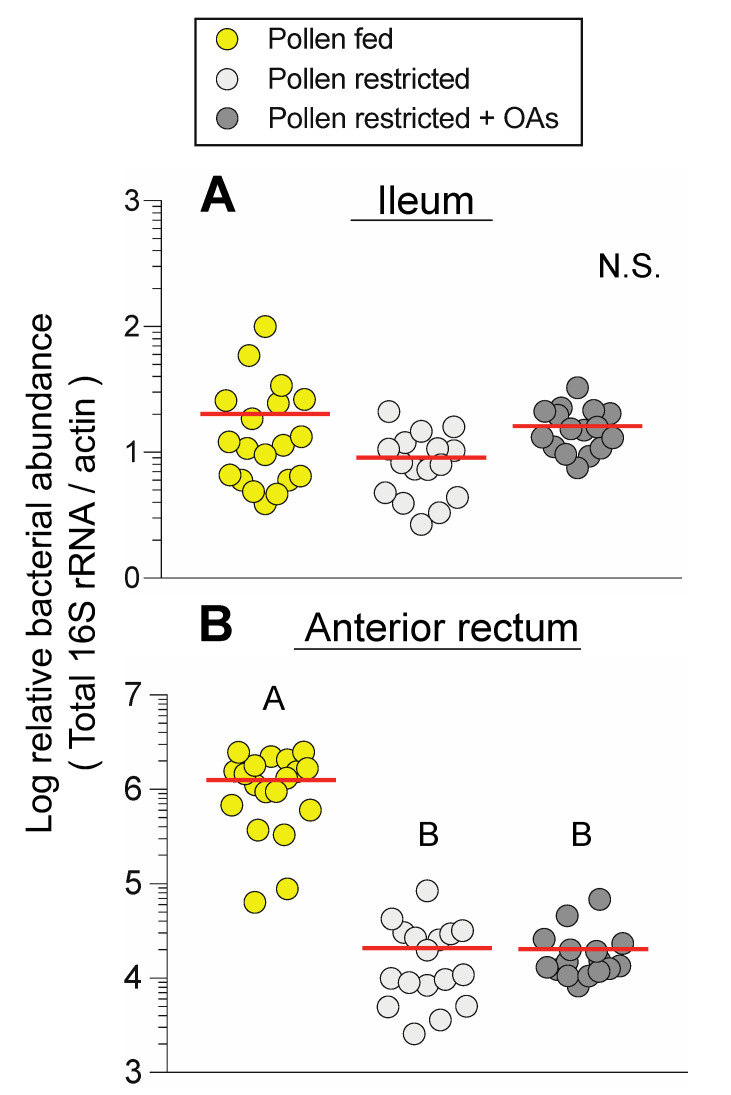
Relative bacterial 16S rRNA abundance in honey bees subjected to different diet treatments (pollen-fed, pollen restricted, pollen restricted + organic acids (OAs)). (**A**) Relative ileum 16S abundance. (**B**) Relative anterior rectum 16S abundance. Each point represents a pooled sample from an independent cage (*n* = 16–19 cages). Black horizontal lines indicate the mean. Different letters indicate Tukey HSD *P* < 0.05.

Three feeding treatments (pollen-fed, pollen-restricted, pollen-restricted + organic acids, OAs) were used to experimentally manipulate diet inputs and measure changes in bacterial abundance and fermentative metabolism as well as honey bee neuropeptide signaling. To first determine the effects of diet manipulations on bacterial abundance, total and specific bacterial 16S copies were measured by qPCR. Total bacterial abundance in the ileum was unaffected by diet treatment (F _2, 48_ = 2.35, *P* = 0.106; Figure 2A). This result was consistent with non-significant changes to specific bacterial abundance in the ileum, although a pollen-induced increase in *Lactobacillus* Firm 4 trended towards significance (Figure S1). Total bacterial abundance in the anterior rectum was significantly reduced by pollen restriction (F _2, 49_ = 41.73, *P* < 0.001; Figure 2B).

**Figure 3:**
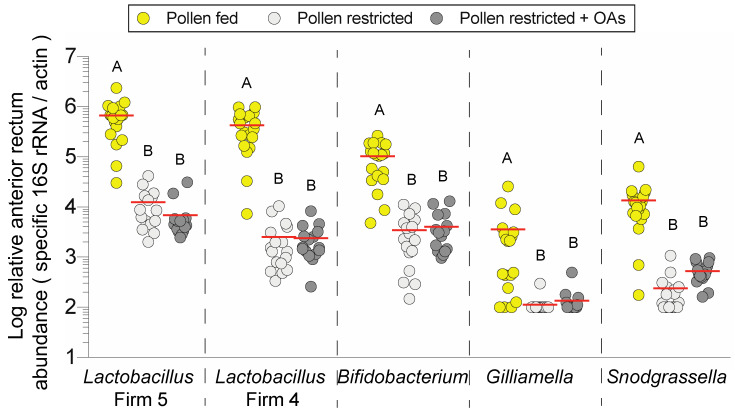
Relative anterior rectum species-specific 16S rRNA abundance in honey bees subjected to different diet treatments (pollen-fed, pollen restricted, pollen restricted + organic acids (OAs)). Each point represents a pooled sample from an independent cage (*n* = 16–19 cages). Black horizontal lines indicate the mean. Different letters indicate Tukey HSD *P* < 0.05.

Consistent with anterior rectum differences in total bacterial abundance, significant species-specific reductions were detected in the treatment groups lacking dietary pollen: *Lactobacillus* Firm 5 (F _2, 49_ = 25.67, *P* < 0.001), and *Lactobacillus* Firm 4 (F _2, 49_ = 33.81, *P* < 0.001), *Bifidobacterium* (F _2, 49_ = 29.13, *P* < 0.001), *Gilliamella* (F _2, 49_ = 5.19, *P* = 0.009), and *Snodgrassella* (F _2, 49_ = 14.68, *P =* 0.003; Figure 3). There were no significant differences in hindgut bacterial abundance between pollen-restricted bees and pollen-restricted bees that were supplemented with organic acids (OAs), although higher ileum abundance of *Snodgrassella* in OA-fed supplemented bees trended towards significance (Figure S1). Overall, dietary pollen restriction significantly reduced anterior rectum bacterial abundance in the nine-day feeding period (Figure 2B and Figure 3).

### 3.3. Bacterial Fermentative Enzyme Gene Transcripts in the Anterior Rectum are Reduced in Association with Diet-Induced Microbiota Shifts

A variety of OAs have been detected in the honey bee gut that is likely attributable to microbiota fermentative processes [9,17], but the influence of diet on microbiota metabolism is largely unknown. Bacterial species-specific primers were used to evaluate the expression of fermentative enzyme genes in response to the different feeding treatments. We monitored the expression of acetate kinase (*ackA*) and L-lactate dehydrogenase (*ldh)* transcripts, which encode enzymes responsible for the production of acetate and lactate, respectively [43]. We also profiled the expression of candidate 3-hydroxybutyryl-CoA dehydrogenase (*hbd*) transcripts putatively involved in butyrate production based on published genome sequences [44] and homology to functionally characterized genes [45,46].

**Figure 4:**
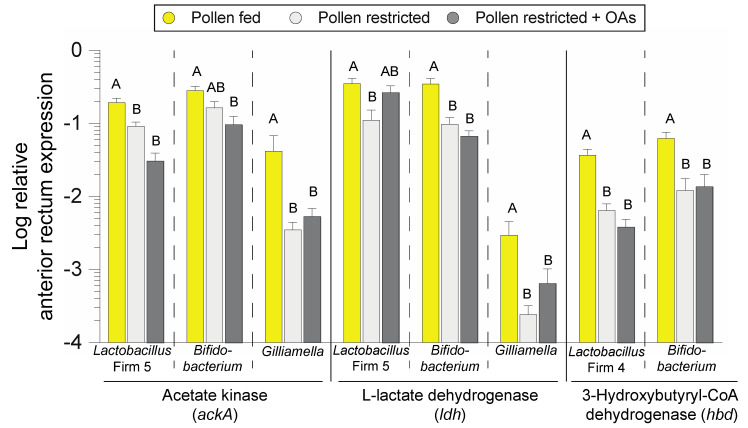
Expression of bacterial fermentative enzyme gene transcripts in the anterior rectum of honey bees subjected to different diet treatments (*n* = 16–19 cages). Error bars represent standard error (SE). Different letters indicate Tukey HSD *P* < 0.05.

Consistent with non-significant changes in ileum bacterial abundance (Figure S1), diet treatment had a minor impact on fermentative gene expression. However, ileum expression of *Lactobacillus* Firm 5 *ackA* was higher in pollen-restricted bees relative to the pollen-restricted + OAs treatment (F _2, 48_ = 3.56, *P* = 0.036) and *Bifidobacterium hbd* expression was highest in pollen fed bees (F _2, 48_ = 11.38, *P* < 0.001; Figure S2). Anterior rectum expression levels of *ackA* were significantly impacted by diet treatment with respect to *Lactobacillus* Firm 5 (F _2, 49_ = 17.75, *P* < 0.001), *Bifidobacterium* (F _2, 49_ = 6.42, *P =* 0.003) but not *Gilliamella* despite trending towards significance (Figure 4). Anterior rectum expression levels of *ldh* were significantly impacted by diet treatment with respect to *Lactobacillus* Firm 5 (F _2, 49_ = 4.47, *P* = 0.017), *Bifidobacterium* (F _2, 49_ = 13.83, *P <* 0.001), but not *Gilliamella* despite trending towards significance. Anterior rectum expression levels of bacterial *hbd* were significantly impacted by diet treatment with respect to *Lactobacillus* Firm 4 (F _2, 49_ = 13.96, *P* < 0.001) and *Bifidobacterium* (F _2, 49_ = 13.83, *P <* 0.001; Figure 4). In general, fermentative enzyme gene expression in the anterior rectum was significantly reduced in the absence of dietary pollen (Figure 4).

### 3.4. Modulation of Enteroendocrine Signaling by Pollen and Organic Acid Feeding

**Figure 5:**
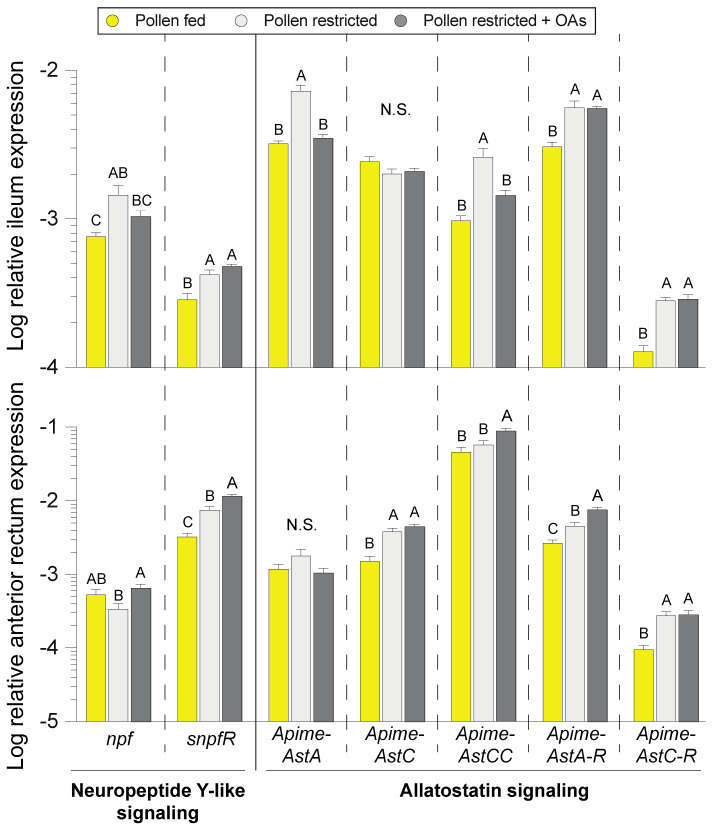
Expression of enteroendocrine signaling gene transcripts in hindgut tissues of honey bees subjected to different diet treatments (*n* = 16–19 cages). Error bars represent standard error (SE). Different letters indicate Tukey HSD *P* < 0.05.

One mechanism by which diet-microbiota interactions can influence the host is through modulation of enteroendocrine signaling by bacterial metabolites. We monitored hindgut expression of honey bee neuropeptide signaling genes that were previously characterized in the brain and were recently identified as part of the enteroendocrine peptide diversity in the bee gut. Due to their nutritional regulation in the bee brain [30], we hypothesized that neuropeptide F (NPF) and allatostatin signaling in the hindgut may respond to metabolites of pollen digestion, including OAs. Transcript expression of the neuropeptide *npf* and its receptor *snpfR*, were significantly impacted by diet treatment in the ileum (*npf*: F _2, 48_ = 5.85, *P =* 0.005; *snpfR*: F _2, 48_ = 12.69, *P <* 0.001) and anterior rectum (*npf*: F _2, 49_ = 3.75, *P =* 0.031; *snpfR*: F _2, 49_ = 39.87, *P <* 0.001; Figure 5). Transcript expression of *npf* was treatment- and gut tissue-dependent whereas *snpfR* was lowest in both gut segments of pollen-fed bees. Expression profiling of allatostatin signaling included the neuropeptide allatostatin A (*Apime-ASTA*) and its receptor (*Apime-ASTA-R*); and C-type allatostatin peptides (*Apime-ASTC* and *Apime-ASTCC)* and their common receptor *Apime-ASTC-R*. Expression of *Apime-ASTA* was significantly impacted by diet treatment in the ileum (F _2, 48_ = 12.69, *P <* 0.001; Figure 5), whereas anterior rectum expression did not differ. Ileum expression of *Apime-ASTA* in the pollen-restricted + OAs treatment group was the same as the pollen-fed group, indicating that OA supplementation could rescue some effects of pollen restriction. Ileum expression of *Apime-ASTC* was not influenced by diet treatment whereas pollen-fed bees had the lowest anterior rectum expression levels (F _2, 49_ = 20.16, *P <* 0.001; Figure 5). *Apime-ASTCC* expression was significantly impacted by diet treatment in the ileum (F _2, 48_ = 14.10, *P <* 0.001) and rectum (F _2, 49_ = 7.34, *P =* 0.002; Figure 5). Ileum expression of *ASTCC* in the pollen-restricted + OAs group recapitulated the pollen-fed group whereas the OAs treatment leads to significantly higher expression levels in the anterior rectum. Expression of *Apime-ASTC-R* in pollen-fed bees was lowest in the ileum (F _2, 48_ = 36.86, *P <* 0.001) and rectum (F _2, 49_ = 12.26, *P <* 0.001; Figure 5).

## 4. Discussion

Here, we describe a putative host-microbiota nutrition interface in the anterior rectum of the honey bee and provide experimental evidence for interactions between diet, microbiota metabolism, and bee enteroendocrine function. Metagenomic and metatranscriptomic analyses have suggested that fermentation is the predominant metabolic function of the gut microbiome [11,15,47]. This was corroborated by metabolomic analyses, which revealed that the microbiota significantly alters metabolite profiles of the gut [9,17]. However, in order to develop nutritional interventions that could eventually improve bee health, an increased understanding of diet-microbiota-host interactions is necessary.

This study employed dietary pollen restriction and OA feeding in an effort to obtain fundamental information about diet-microbiota-host interactions in honey bees. Experimental manipulations of bee gut bacteria typically employ whole guts, although subsectioning into separate gut regions (midgut, ileum, and rectum) can provide increased resolution of treatment effects [12,40]. We first tested the hypothesis that the rectum bacterial community is at least partially associated with the epithelium of the anterior portion of this gut region. The bee anterior rectum features rectal pads (Figure 1), which are specialized epithelial structures of the insect hindgut that extract water and ions from the lumen [48,49]. While the function of rectal pads in honey bees is not fully understood, it is possible that these structures regulate hindgut physiochemical conditions that support microbiota growth and fidelity as well as host-microbe cross-talk. Quantification of total bacterial 16S abundance revealed 10^8^ DNA copies in the anterior rectum after it had been washed of lumen contents (Figure 1). This result is consistent with a biofilm in the upstream ileum, which harbors a distinct community that expresses approximately 10^7^ bacterial 16S copies [10]. Hence, the anterior rectum and ileum were used as target tissues to test the effects of diet manipulations on the bee host and microbiota.

Despite its simple structure and the limited number of bacterial species, the bee microbiota exhibits high strain diversity, which may act as a reservoir of metabolic functions that modulate nutrition [50]. Different lines of evidence collectively indicate that the bee microbiota ferments pollen fibers, although the impact of pollen diet on bacterial abundance is comparatively less understood. We found that pollen restriction markedly decreased total bacteria in the anterior rectum but not the ileum (Figure 2). This was further examined using species-specific 16S rRNA primers, which confirmed that pollen alters fermentative bacteria abundance in the anterior rectum (Figure 3). The rectum community is dominated by the fermentative groups of *Lactobacillus* Firm 4 and Firm 5 and *Bifidobacterium,* whereas the ileum harbors a biofilm of the non-fermentative *Snodgrassella,* which participates in syntropic (nutrient-sharing) interactions with fermentative community members such as *Gilliamella* [12,17].

While our understanding of host-microbiota interactions in the honey bee is still in its infancy, many hypotheses can be gleaned from more established systems such as human-gut microbiota interactions. The fermentation of dietary fiber into organic acids involves a number of reactions mediated by the enzymatic repertoire of fermentative gut bacteria [40]. While the primary fermentation products of indigestible fiber are typically acetate, propionate, and butyrate [51], the honey bee microbiota also produces lactate, succinate, and formate [17,43]. All of these OAs have direct metabolic roles as well as conserved roles in gut epithelium function [38,51]. Consistent with diet-induced microbiota shifts, pollen restriction markedly reduced fermentative enzyme gene expression (Figure 4). Although *Snodgrassella* utilizes carboxylates, including OAs for growth [14], feeding OAs did not influence the abundance of this non-fermentative bacterium, despite it trending towards increased abundance in the ileum (Figure S1). We used taxon-specific primers to measure the expression of *ackA* and *ldh*, which encode enzymes responsible for the production of acetate and lactate, respectively. These primer sets were previously used to compare transcript levels across gut compartments (crop, midgut, hindgut), indicating that the hindgut is the primary site of bacterial gene expression [43]. Sectioning of the hindgut into the ileum and anterior rectum revealed that *ackA* and *ldh* are predominantly expressed in the anterior rectum and are regulated by dietary pollen (Figure 4) but not the addition of OAs. We also monitored the expression of genes that catalyze the final step to butyrate. Based on sequence homology to functionally characterized genes, primers were designed to target *Lactobacillus* Firm 4 and *Bifidobacterium hbd*, a critical enzyme involved in directing carbon flux towards butyrate production, particularly during fermentation of complex carbohydrates. Similar to *ackA* and *ldh* transcript levels, the expression of *hbd* was markedly reduced in the absence of dietary pollen (Figure 4). These results highlight the role of the pollen in gut bacteria metabolism *in vivo* and the utility of qPCR-based metatranscriptomic expression profiling to monitor community-level metabolic changes. Further, expression of fermentative metabolism may prove to be a useful biomarker in characterizing the impact of non-pollen diets on the honey bee microbiota with the aim of recapitulating metabolic profiles of pollen-fed bees [52].

In arthropods, a number of tissues produce peptide hormones that exert modulatory control of physiology and behavior, the most well-characterized being the nervous system and its associated neuroendocrine organs [20,23,53]. Epithelial enteroendocrine cells are a major site of peptide hormone production and secretion, often operating via the same receptors and cellular transduction mechanisms as their neural equivalents [18,19]. In other host-microbe systems, organic acids also act as specific G-coupled protein receptor (GCPR) signaling molecules [21]. Thus, the microbiota can influence its host by modulating enteroendocrine signaling. The bee microbiota has been shown to impact hormone signaling but specific mechanisms by which this occurs are unclear [9]. Further, species-specific increases in core *Lactobacillus* and *Bifidobacterium* in the rectum of long-lived queens have been suggested to modulate host physiology and health through fermentative metabolites such as butyrate [54]. Under the different feeding conditions, we profiled the hindgut expression of neuropeptide signaling pathways that were recently identified as part of the enteroendocrine diversity in the bee gut [25]. Neuropeptide F (NPF) and allatostatin signaling genes exhibited treatment- and tissue-specific expression patterns, indicating their potential involvement in hindgut nutrient sensing and signal transduction (Figure 5). In general, the pollen diet led to the downregulation of neuropeptide signaling genes whereas pollen restriction led to their upregulation. Intriguingly, the pollen-restricted + OAs treatment could partially rescue the expression phenotype of pollen-fed bees in some cases. For instance, ileum transcript expression of allatostatin peptides *Apime-AstaA* and *Apime-ASTCC* were highest in pollen-restricted bees while the OAs treatment mirrored pollen-fed bees. Although not significant, *npf* expression in the anterior rectum trended towards higher levels in pollen-restricted + OAs bees relative to pollen-restricted bees. The insect NPF pathway is a close homolog of the mammalian neuropeptide Y pathway, which is well-characterized to be involved in the regulation of hunger as well as various physiological and homeostatic processes [27]. Consistent with previously reported expression patterns in the brain, gut expression of *snpfR*, the only NPF-family receptor encoded by the honey bee genome [26], was upregulated by pollen restriction.

## 5. Conclusions

Elucidation of mechanisms by which the gut microbiota interacts with nutrition could lead to dietary interventions to improve honey bee health. Here, we showed that the bacterial community of the honey bee gut is partially associated with the epithelium of the anterior rectum. This gut region warrants further investigation as a potential site of luminal chemosensing or metabolic exchange with the gut microbiota, particularly due to its high bacterial abundance, proximity to the ileum, and the presence of host rectal pads. Improvements in our approach should aim to employ fine-scale tissue sectioning in order to characterize the total amount of gut wall-associated bacteria versus lumen bacterial abundance within the rectum. Pollen restriction and OA feeding provided insights on the interaction between diet, microbiota fermentation products, and the honey bee host. Additional investigations are required to further elucidate the underlying mechanisms of diet-based microbiota influence on the honey bee.

## Supplementary Materials

The following are available online at https://www.mdpi.com/2075-4450/11/5/291/s1, Figure S1: Relative ileum species-specific 16S rRNA abundance in honey bees subjected to different diet treatments., Figure S2: Expression of bacterial fermentative enzyme gene transcripts in the ileum of honey bees subjected to different feeding treatments, Table S1: Primers used in this study.
