# Supplementary material for: Probing the Honey Bee Diet-Microbiota-Host Axis Using Pollen Restriction and Organic Acid Feeding
Source: Insects. 2020 May 9;11(5):291. doi: 10.3390/insects11050291 (PMC7291221; doi:10.3390/insects11050291)
Supplement: Supplementary file 1 [file insects-11-00291-s001.pdf]

insects

MDPI

Table S1. Primers used in this study.

**Honey bee**<br>**Gene**	**Forward 5′-3′**	**Reverse 5′-3′**	**Annealing temperature (°C)**	**Reaction efficiency (logarithmic dilution)**	**Study**
actin	TGCCAACACTGTCCTTTCTG	AGAATTGACCCACCAATCCA	55.0	1.01	[1]
npf	GAACTTTTGGGTGAGCACGAA	ACGTGACAAGGTCGAGTATCGA	60.0	0.94	[2]
snpfR	AATTTGGCATTGAGCGATGTT	CCGAGGAACGTGTACAATGGT	60.0	0.92	[2]
AstA	GTCTCCGAGTATAAGAGGCTACC	TGTATTGTCTTCGTTTACCAAGCC	55.0	0.98	[3]
AstC	CCTGTTCACGAAACAAATCG	TATTTGCCAAAGCAAGAGA	55.0	1.04	[3]
AstCC	GAGCAACAAACGHTAAAGATGG	TTCTCTTGAAACACGTCACAG	55.0	0.89	[3]
AstAR	CGACGATAATGGTGC	CTAAATGATGTCACCGG	55.0	1.13	[3]
AstCR	CGAGTTTTAGGACRAGGAC	TCTGCTCCTTCGGCTCCATTG	55.0	0.90	[3]
**Microbiota**<br>**Gene**	**Forward 5′-3′**	**Reverse 5′-3′**	**Annealing temperature (°C)**		**Study**
Lactobacillus Firm 5 16S rRNA	GCAACCTGCCCTWTAGCTTG	GCCCATCCTKTAGTGACAGC	60.0	0.98	[4]
Lactobacillus Firm 4 16S rRNA	AGTCGAGCGCGGGAAGTCA	AGCCGTCTTTCAACCAGCACT	60.0	0.97	[4]
Bifidobacterium 16S rRNA	ATGCAAGTCGAACGGGATCC	CATCCCATRCCGGTAAACCC	60.0	0.99	[4]
Gilliamella 16S rRNA	CTTTGTTGCCATCGGTTAGGCC	CCGCTTGCTCTCGCGAGG	60.0	0.89	[5]

Snodgrassella 16S rRNA	CTTAGAGATAGGAGAGTGCCTT	AACTTAATGATGGCAACTAATGACAA	60.0	1.08	[4]
Lactobacillus Firm 5 ackA	GGTGTTCCTGAAGTAGGCGT	GAGCAGCGCGTCCTACAATA	62.0	0.95	[6]
Bifidobacterium ackA	GGCGAAAACCGTCCTAGTCA	CGGTCTCATGGTCGTGGATG	62.0	1.18	[6]
Gilliamella ackA	GCGCCATAACGACGAATACC	TCGTGAAGCTTTTGCCGAGT	62.0	0.87	[6]
Lactobacillus Firm 5 ldh	GTTGTTGTGATCACCGCTGG	AGGGAAGTACCTGTCCCAAT	62.0	0.90	[6]
Bifidobacterium ldh	AAGGGGTCAACACCCACATC	TTGCTTGCCGTCTCCTTGAG	62.0	1.21	[6]
Gilliamella ldh	TAGCTTGCGCGAATCAACTG	CAATCACACGGTGACGAGGT	62.0	0.93	[6]
Lactobacillus Firm 4 hbd	ATCGATGTGACCGTGGCTTT	CATCAACGGCCTGAGGAGAA	60.0	0.88	This study
Bifidobacterium hbd	TCGATGCTGGGCTGAACAAT	TTGCTGGCGAAGATGGTCTT	60.0	0.91	This study

References:

1. Alaux, C.; Dantec, C.; Parrinello, H.; Le Conte, Y. Nutrigenomics in honey bees: digital gene expression analysis of pollen's nutritive effects on healthy and varroa-parasitized bees. *BMC Genomics* **2011**, *12*, 496.
2. Ament, S. A.; Velarde, R. A.; Kolodkin, M. H.; Moyse, D.; Robinson, G. E. Neuropeptide Y-like signalling and nutritionally mediated gene expression and behaviour in the honey bee. *Insect Mol. Biol.* **2011**, *20*, 335–345.
3. Urlacher, E.; Soustelle, L.; Parmentier, M.-L.; Verlinden, H.; Gherardi, M.-J.; Fourmy, D.; Mercer, A. R.; Devaud, J.-M.; Massou, I. Honey Bee Allatostatins Target Galanin/Somatostatin-Like Receptors and Modulate Learning: A Conserved Function? *PLoS ONE* **2016**, *11*, e0146248–30.
4. Kešnerová, L.; Mars, R. A. T.; Ellegaard, K. M.; Troilo, M.; Sauer, U.; Engel, P. Disentangling metabolic functions of bacteria in the honey bee gut. *PLoS Biol* **2017**, *15*, e2003467.
5. Emery, O.; Schmidt, K.; Engel, P. Immune system stimulation by the gut symbiont Frischella perrara in the honey bee (Apis mellifera). *Mol Ecol* **2017**, *26*, 2576–2590.
6. Lee, F. J.; Miller, K. I.; McKinlay, J. B.; Newton, I. L. G. Differential carbohydrate utilization and organic acid production by honey bee symbionts. *FEMS Microbiology Ecology* **2018**, *94*, 3557.

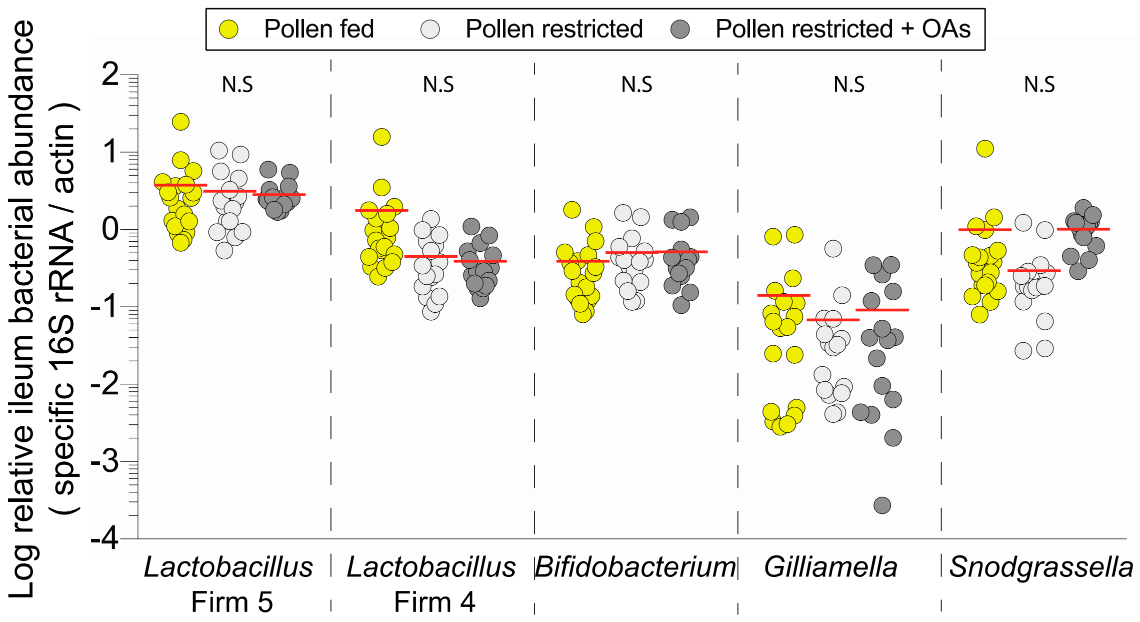

Figure S1. Relative ileum species-specific 16S rRNA abundance in honey bees subjected to different diet treatments. Each point represents a pooled sample from an independent cage (n = 16–19 cages). Black horizontal lines indicate the mean. No significant differences were detected. .

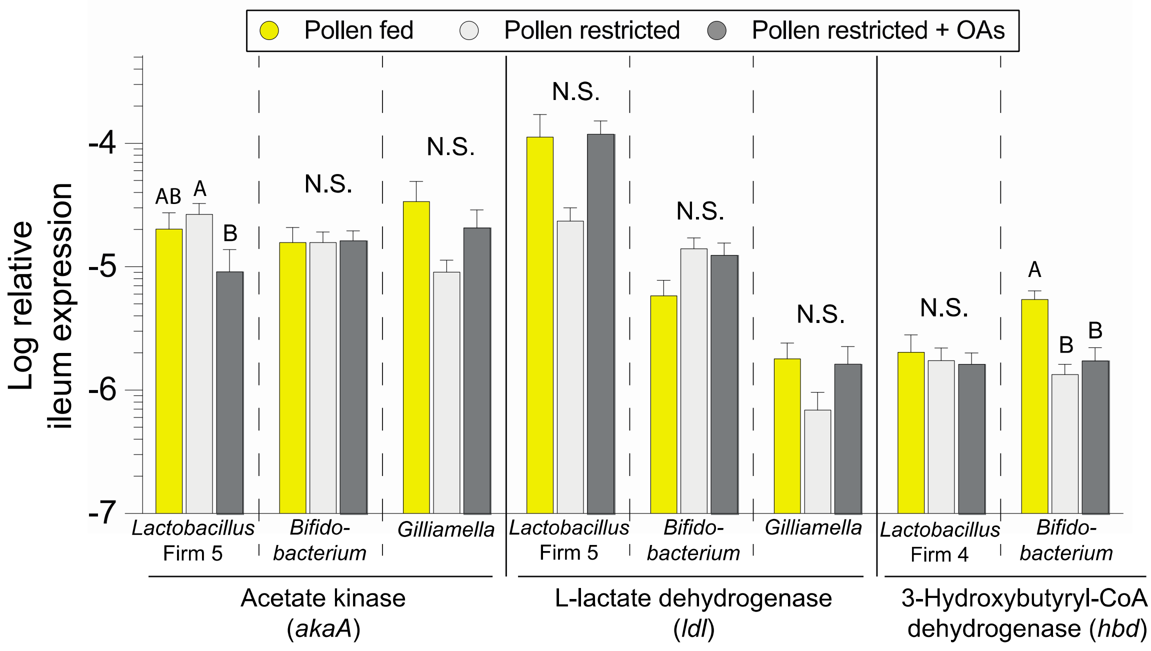

Figure S2. Expression of bacterial fermentative enzyme gene transcripts in the ileum of honey bees subjected to different feeding treatments (n = 16–19 cages). Error bars represent standard error (SE). Different letters indicate Tukey HSD $P < 0.05$.

© 2020 by the authors. Licensee MDPI, Basel, Switzerland. This article is an open access article distributed under the terms and conditions of the Creative Commons Attribution (CC BY) license (http://creativecommons.org/licenses/by/4.0/).
